# Bone wax-tipped catheter and 3-way stopcock to optimize hemostatic powder deployment

**DOI:** 10.1016/j.vgie.2021.05.011

**Published:** 2021-06-23

**Authors:** J. Andy Tau, Zaid Imam, Fateh Bazerbachi

**Affiliations:** 1 Austin Gastroenterology PA, Austin, Texas; 2 Division of Gastroenterology, Department of Medicine, William Beaumont Hospital, Royal Oak, Michigan; 3 CentraCare, Interventional Endoscopy Program, St. Cloud Hospital, St. Cloud, Minnesota

## Abstract

Video 1 Preparation and demonstration of bone wax–tipped catheter for optimized hemostatic powder delivery ex vivo and in vivo.

Preparation and demonstration of bone wax–tipped catheter for optimized hemostatic powder delivery ex vivo and in vivo.

## Introduction

TC-325 Hemospray (Cook Medical, Winston-Salem, NC, USA) is a highly absorptive mineral powder used for endoscopic hemostasis in the setting of nonvariceal upper GI bleeding. The hemostatic powder is propelled by compressed CO_2_ through either a 7F or 10F catheter inserted through the working channel of the endoscope.

On contact with blood, the hemostatic powder rapidly absorbs water, producing a mechanical barrier and causing thrombus formation by concentrating platelets and clotting factors.^1^ This agglomerative property, however, poses a challenge when propelling the powder through the working channel of the endoscope, which is also the conduit for suction, because the powder rapidly occludes a wet delivery catheter. This is particularly an issue for the 7F catheter. Although the 10F catheter is less prone to occlusion, it only fits in a therapeutic channel gastroscope or adult colonoscope.

Thus, 3 technical limitations arise that are particularly compounded during active, refractory bleeding (rescue/salvage therapy) when intralumenal blood is unavoidable:

1. The working channel must be dried by flushing air at the biopsy port. Flushing the catheter itself during passage down the instrument channel has also been suggested.^2^
2. The catheter tip cannot get wet.
3. Suction is strictly prohibited.

## Procedure

We propose the simple application of a hydrophobic bone wax plug, which seals the tip of the delivery catheter, resolving all 3 limitations at once. One can skip drying the working channel, allow the tip of the catheter to freely contact blood, and even use suction.

Bone wax is a safe, sterile, nonabsorbable mixture of beeswax and isopropyl palmitate that provides an impermeable hydrophobic seal. It has been used to endoscopically deploy fiducials in malignant lesions, preventing introduction of air, which impedes ultrasound visualization.^3^ It also has precedent for use in bronchoscopy brushes to protect against fluid contamination.^4^

**Figure 1:**
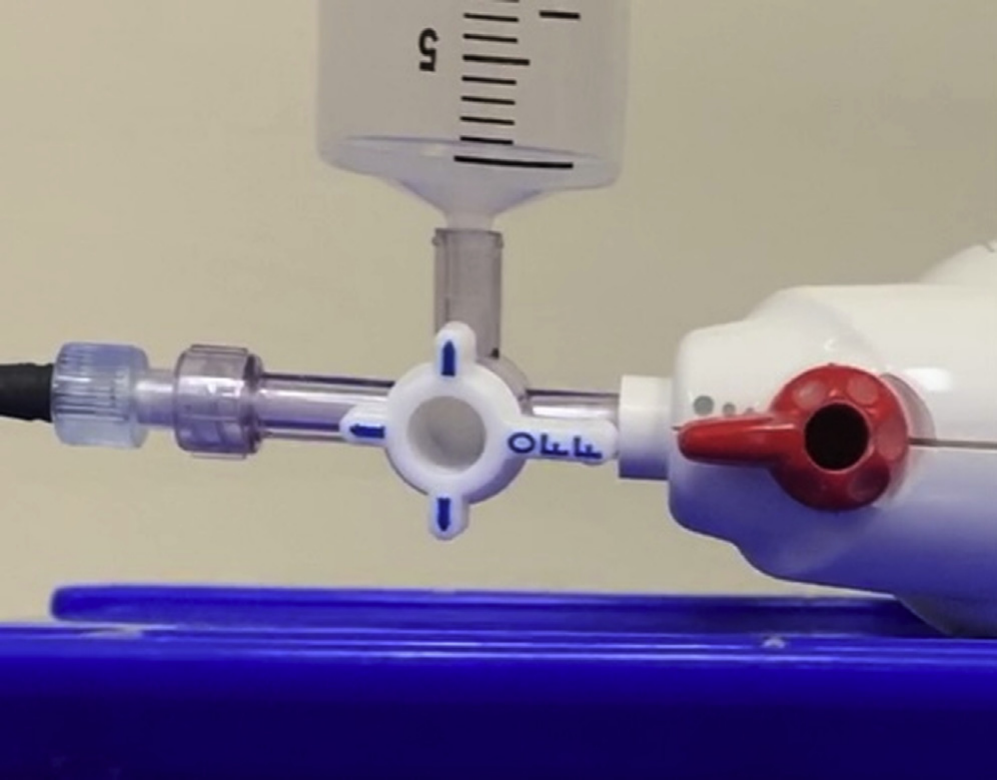
Eject bone wax with stopcock-opposing device.

A small amount of bone wax is shaped into a bead and applied to the tip of the 7F or 10F delivery catheter. The catheter is then affixed to the Luer-lok end of a 3-way stopcock. The activated device and an air-filled syringe are attached to the remaining ends of the stopcock. The stopcock is turned to oppose the device (Fig. 1)

**Figure 2:**
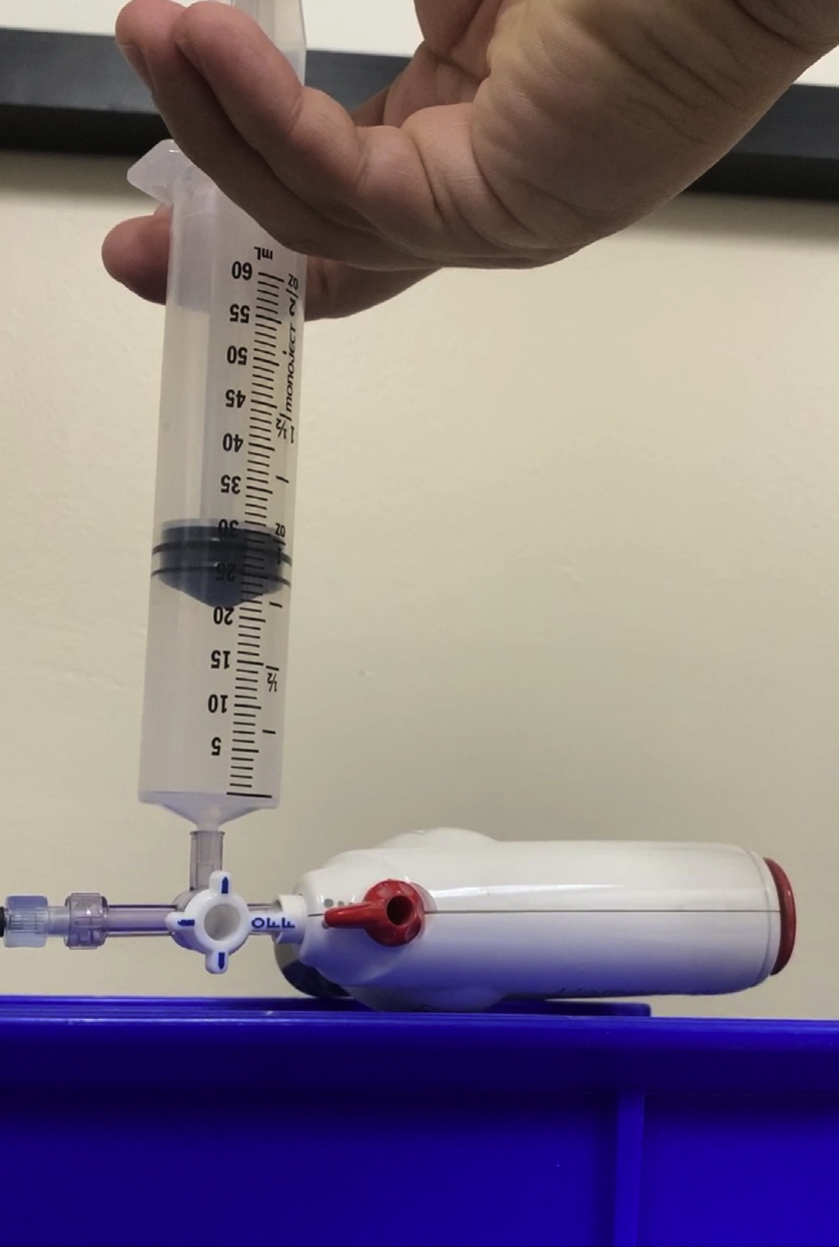
Air flush from prefilled syringe to eject bone wax.

**Figure 3:**
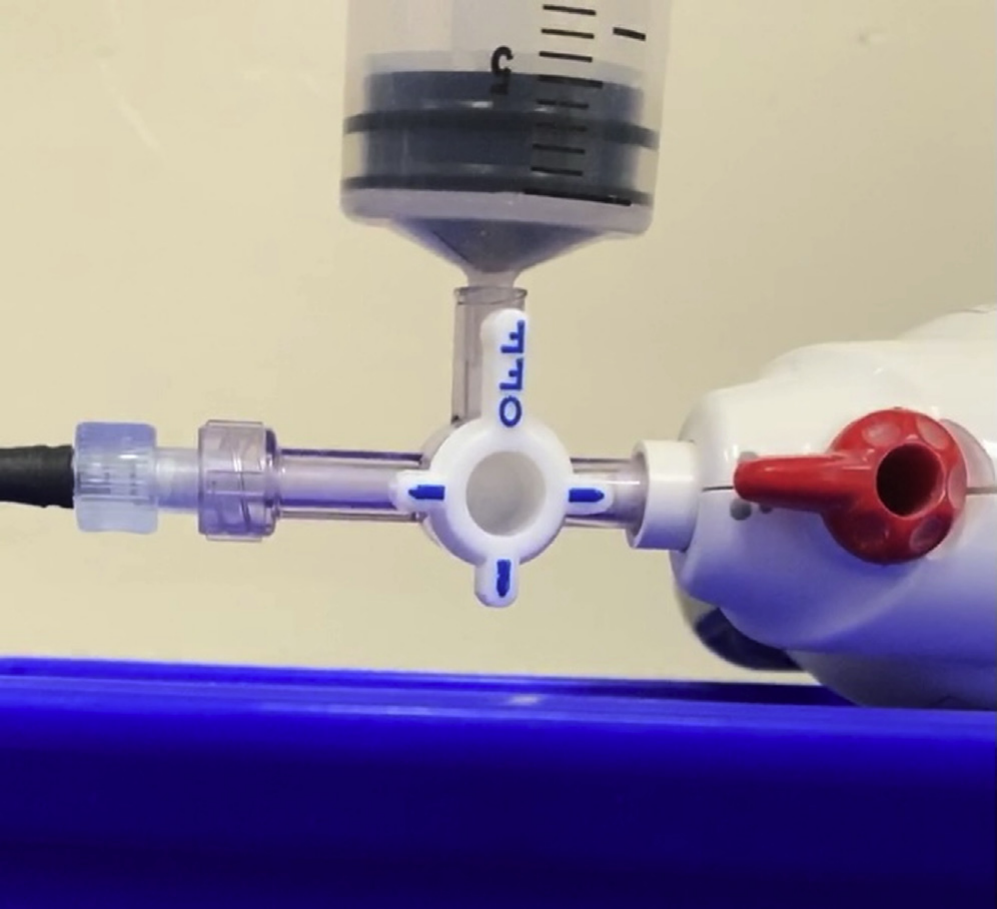
Deliver powder with stopcock opposing syringe.

Without having to dry the working channel or air flush the catheter, the bone wax–tipped catheter is passed down the channel in standard fashion. With this technique, the catheter tip can get wet, and fluid can be suctioned, particularly around the 7F catheter. When ready to fire, the bone wax plug is flushed off using air from the prefilled syringe (Fig. 2), the stopcock is turned to oppose the syringe (Fig. 3), and the hemostatic powder is deployed, all in rapid sequence.

We demonstrate the preparation and application of this technique in both ex vivo and multiple in vivo cases (Video 1, available online at www.giejournal.org).

## Discussion

We have applied this method in 41 cases (31 cases with 7F and 10 cases with 10F catheters). Successful hemostasis without catheter obstruction was achieved in all cases, without requiring a second catheter or the preparatory drying/flushing steps suggested by the manufacturer. No adverse events related to the application of bone wax were encountered in any of our cases. Because catheter clogging is no longer an issue, we now prefer the 7F catheter combined with a therapeutic channel gastroscope to maximize suction capacity.

**Figure 4:**
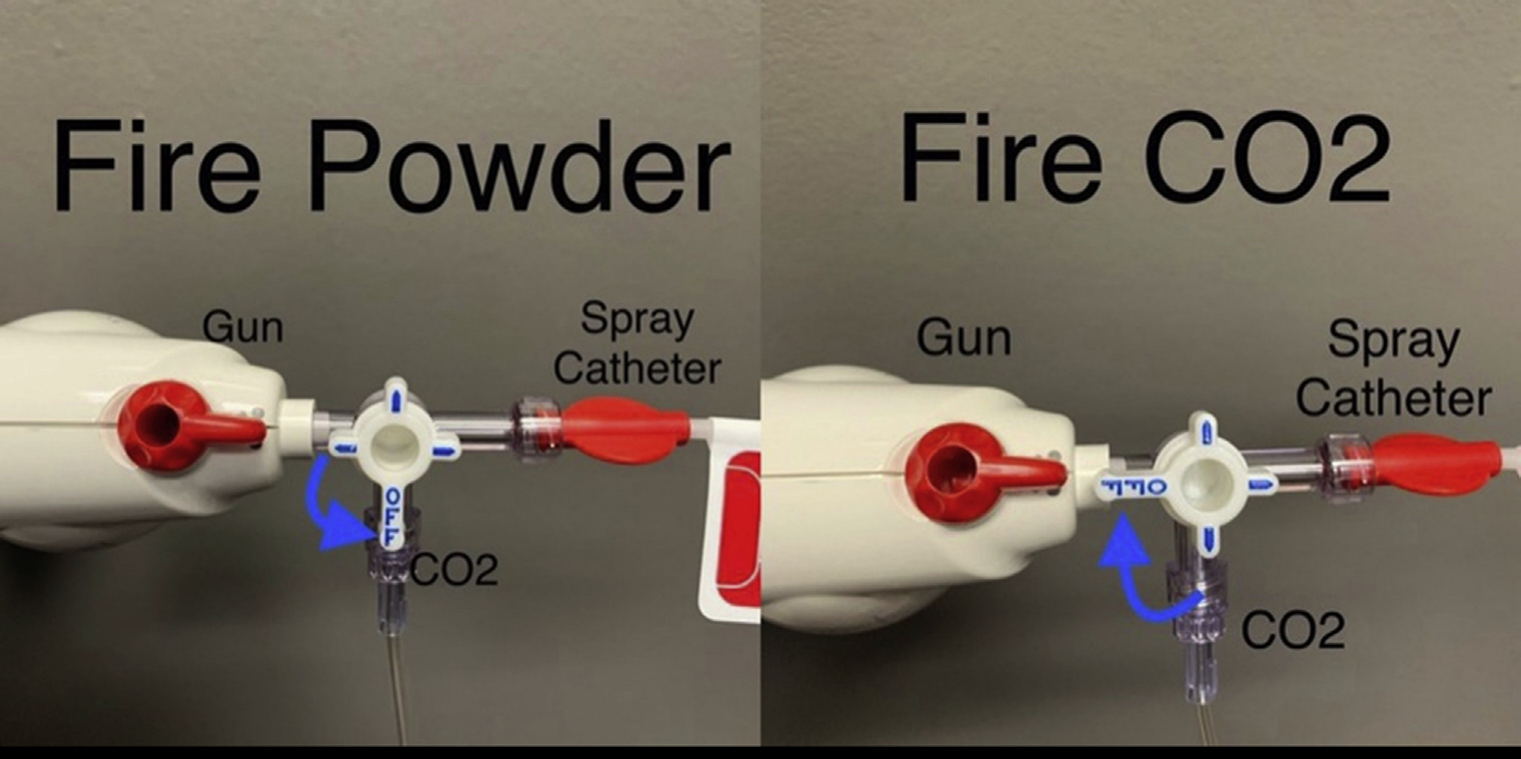
Use continuous stream of CO_2_ to prevent catheter occlusion after bone wax ejection.

Admittedly, after the protective bone wax plug is ejected, the catheter tip is exposed and can be occluded if wet. Air flushes from the syringe through the stopcock can help rapidly clear the occlusion, and then one can quickly switch back to powder delivery with a turn of the stopcock. Alternatively, rerouting the CO_2_ cannula from the endoscope onto the 3-way stopcock, in lieu of the air-filled syringe, can provide a continuous protective stream of CO_2_ after the bone wax is ejected.^5^ The technician can nimbly turn the stopcock, switching seamlessly between powder delivery and a protective stream of CO_2_ (Fig. 4). The endoscope can still use room air at the push of a button, if needed.

## Conclusions

Hemostatic powder is often used for salvage hemostasis in refractory hemorrhage, but because of the nature of the bleeding and the powder, catheter occlusion can be a significant obstacle. Our method using a bone wax–tipped catheter protects the catheter from fluid, eliminates preparatory air flushing, and allows for suctioning of blood before initial powder application. This method is simple and cheap and effectively optimizes the use of this life-saving device.

## Supplementary data

Video 1 Preparation and demonstration of bone wax–tipped catheter for optimized hemostatic powder delivery ex vivo and in vivo.
